# Isolation of *Mycobacterium avium* Subspecies *paratuberculosis* Reactive CD4 T Cells from Intestinal Biopsies of Crohn's Disease Patients

**DOI:** 10.1371/journal.pone.0005641

**Published:** 2009-05-22

**Authors:** Ingrid Olsen, Stig Tollefsen, Claus Aagaard, Liv J. Reitan, John P. Bannantine, Peter Andersen, Ludvig M. Sollid, Knut E. A. Lundin

**Affiliations:** 1 Department of Animal Health, National Veterinary Institute, Oslo, Norway; 2 Centre for Immune Regulation, Institute of Immunology, University of Oslo and Rikshospitalet University Hospital, Oslo, Norway; 3 Department of Infectious Disease Immunology, Statens Serum Institut, Copenhagen, Denmark; 4 National Animal Disease Center, USDA-Agricultural Research Service, Ames, Iowa, United States of America; 5 Department of Medicine, Rikshospitalet University Hospital, Oslo, Norway; New York University School of Medicine, United States of America

## Abstract

**Background:**

Crohn's disease (CD) is a chronic granulomatous inflammation of the intestine. The etiology is unknown, but an excessive immune response to bacteria in genetically susceptible individuals is probably involved. The response is characterized by a strong Th1/Th17 response, but the relative importance of the various bacteria is not known.

**Methodology/Principal Findings:**

In an attempt to address this issue, we made T-cell lines from intestinal biopsies of patients with CD (n = 11), ulcerative colitis (UC) (n = 13) and controls (n = 10). The T-cell lines were tested for responses to various bacteria. A majority of the CD patients with active disease had a dominant response to *Mycobacterium avium* subspecies *paratuberculosis* (MAP). The T cells from CD patients also showed higher proliferation in response to MAP compared to UC patients (p<0.025). MAP reactive CD4 T-cell clones (n = 28) were isolated from four CD patients. The T-cell clones produced IL-17 and/or IFN-γ, while minimal amounts of IL-4 were detected. To further characterize the specificity, the responses to antigen preparations from different mycobacterial species were tested. One T-cell clone responded only to MAP and the very closely related *M. avium* subspecies *avium* (MAA) while another responded to MAP, MAA and *Mycobacterium intracellulare*. A more broadly reactive T-cell clone reacted to MAP1508 which belongs to the esx protein family.

**Conclusions/Significance:**

The presence of MAP reactive T cells with a Th1 or Th1/Th17 phenotype may suggest a possible role of mycobacteria in the inflammation seen in CD. The isolation of intestinal T cells followed by characterization of their specificity is a valuable tool to study the relative importance of different bacteria in CD.

## Introduction

Crohn's disease (CD) is an intestinal disorder characterized by granulomatous inflammation. The etiology is still unknown, but it is generally believed that an inappropriate inflammatory response to the commensal bacteria is involved [1]. Lately it has become clear that the risk of developing CD is associated with polymorphisms in several genes that are involved in interaction with bacteria. In particular, NOD2 [2], [3], which is an intracellular sensor of bacteria, and ATG16L1 [4] and IRGM [5], which are involved in autophagy, are genetic factors for CD. NOD2 activates an NF-κB signaling pathway upon binding of the bacterial peptidoglycan component muramyl dipeptide (MDP), but exactly how NOD2 is involved in CD has not been settled. There is evidence both for loss and gain of functions [6], [7]. Autophagy, with involvement of ATG16L1 and IRGM, is an important constitutive cellular process involved in protein turnover and the removal of subcellular components. Recently ATG16L1 was shown to be important for the biology of intestinal Paneth cells [8], and interestingly the autophagy pathway is also important for resistance against intracellular bacteria [9]. Functional knock down of ATG16L1 abrogated autophagy of the intracellular pathogen *Salmonella typhimurium* [10]. Moreover, knockdown of IRGM leads to markedly prolonged survival of *Mycobacterium tuberculosis* in human macrophages [11]. It is notable that *NOD2*, *ATG16L1* and *IRGM* are all risk factors for CD but not ulcerative colitis (UC), while many other genes including the *IL-23 r* gene and the *IL-12B* gene [12] , coding for the common p40 subunit of IL-12 and IL-23, are susceptibility determinates for both conditions. This indicates that some of the inflammatory pathways are likely shared between the two conditions, while the importance of immune handling of bacteria differentiates CD pathophysiology from UC.

At this stage it is unclear whether the CD associated variants of *NOD2*, *ATG16L1* and *IRGM* influence the host response to particular bacteria or whether they have more general effects to a wide range of gut bacteria. Several bacteria have been suggested to be involved in CD pathogenesis including *Escherichia coli* and *Mycobacterium avium* subspecies *paratuberculosis* (MAP). Invasive *E. coli* have been found in higher frequencies in ileal CD [13]. The data on the presence of MAP are not uniform, but two meta-analysis of several published studies have concluded that MAP is more often present in CD patients than patients with UC and non-inflammatory bowl disease (non-IBD) [14], [15]. However whether the bacterium can contribute to the inflammatory response is not known.

The CD lesions are transmural, and typically they have granulomas and lymphoid aggregates with abundance of CD4+ T cells that produce inflammatory cytokines like IL-17 and IFN-γ [16]. To get more information about the bacteria involved in CD pathogenesis one approach is to isolate intestinal T cells. Studies of the specificity of intestinal T-cells in CD are limited. A decade ago Duchman et al showed that both CD and ulcerative colitis (UC) patients had T cells with reactivity to various commensal bacteria, including *E. coli,* however no differences were found between the two groups [17], [18]. To get information about the relative importance of various bacteria in the ability to elicit an inflammatory T cell response, we chose to characterize the specificity of intestinal T cells from CD patients. We subsequently isolated T cells from intestinal biopsies of CD, UC and non-IBD patients and detected responses to all the tested bacteria. However, CD patients had a higher frequency of MAP reactive T cells than the UC patients and also a higher frequency of response to MAP compared to other bacterial antigens. Furthermore these T cells produced inflammatory cytokines like IFN-γ and IL-17. Our data justify further studies into the possible role of mycobacteria in CD immunopathology.

## Methods

### Study subjects

Intestinal biopsies were obtained by colonoscopy from adult patients with CD (n = 11), UC (n = 13) and non-IBD (n = 10). The colonoscopy was performed as a part of the routine investigation. Patients with endoscopically active and inactive disease were included. Patients that had received, or were receiving anti-TNF-α treatment, were not included. Of the CD patients (2 men, 9 women), four had inactive while seven had active disease. The average age was 45 years (range 27–66) and the average time since diagnosis was 19 years (range 5–28). Of the UC patients (8 men, 5 women), five had inactive disease while eight had active disease. The average age was 41 years (range 19–61) and the average time since diagnosis was 12 years (range 2–30). The average age of non-IBD patients (3 men, 7 women) was 49 years (range 18–73). Information about disease localization, medication and diagnosis is given in table 1. All patients gave written informed consent before the colonoscopy. The study was approved by the Regional Committee for Medical Research Ethics, South Norway, and approval for storing of biological materials was obtained by the Norwegian Directorate for Health and Social Affairs.

**Table 1:** Patient characteristics

CD	Disease localisation and actvity	Medication
CD-6	Small intestine (inactive)	Azathioprin
CD-9	Small intestine and colon (active)	Topical steroids
CD-10	Colon (active)	Prednisolone
CD-11	Small intestine (inactive)	None
CD-15	Colon, fistulas (active)	None
CD-18	Small intestine and colon (active)	Mesalazine, Budesonide CR, Colestyramine
CD-33	Colon (inactive)	Azathioprin
CD-36	Colon (inactive)	None
CD-46	Ileocecal and perianal (active)	None
CD-47	Small intestine, ileocecal and perianal (active)	Azathioprin
CD-48	Colon (active)	Balsalazide, Prednisolone

UC	Disease localisation and actvity	Medication
UC-14	Pancolitis (active)	Azathioprin, Balsalazide, Budesonide CR
UC-16	Proctitis (active)	Mesalazine topical
UC-17	Pancolitis (inactive)	Mesalazine, Prednisolone, Tacrolimus
UC-19	Pancolitis (inactive)	Mesalazine
UC-25	Proctitis (inactive)	None
UC-27	Pancolitis (active)	Prednisolone
UC-31	Pancolitis (active)	None
UC-32	Pancolitis (active)	Balsalazide
UC-35	Pancolitis, inactive)	Balsalazide
UC-37	Pancolitis (inactive)	None
UC-40	Pancolitis (active)	Mesalazine
UC-44	Proctitis (active)	Mesalazine topical
UC-45	Pancolitis (active)	Mesalazine

non IBD	Diagnosis	Medication
non IBD-20	Diarrhea	None
non IBD-23	Healthy, family history of colon cancer	None
non IBD-24	Cancer control	Levotyroksin, allergy medication
non IBD-26	Familial adenomatous polyposis	None
non IBD-28	Healthy, family history of colon cancer	None
non IBD-30	Cancer control	None
non IBD-34	Healthy, family history of colon cancer	None
non IBD-13	Primary sclerosing cholangitis, no sign of UC or CD	None
non IBD-49	Healthy, family history of colon cancer	None
non IBD-51	Celiac disease	None

### HLA-typing

The patients were genomically HLA typed using the Olerup SSP HLA kits for DQB1*, DRB1*, DPB1* (GenoVision/Qiagen) or serologically typed by a complement dependent cytotoxicity test with immunomagnetically separated cells (Dynabeads® HLA class II, Invitrogen).

### Strains and antigens

The following strains were used to prepare the antigens: *Bacterioides thetaiotaomicron* CCUG 12297, *Lactobacillus gasseri* CCUG 39972, *Bifidobacterium bifidum* CCUG 45217, *Escherichia coli* ATCC 43893 (enteroinvasive), *M. avium* subsp. *paratuberculosis* 2E, *Mycobacterium avium* subspecies *avium* D4, *Mycobacterium intracellulare* MNC72, *Mycobacterium gordonae* MNC 64, *Mycobacterium tuberculosis* clinical isolate. The bacteria were grown on standard medium under recommended conditions. The cells were scraped off the agar plates, sonicated (two cycles of 10 min) and centrifuged. The supernatants were sterile filtered (0.2 µm) before the protein concentration was assessed according to Lowry [19] using the Bio-Rad D_C_ Protein Assay (Bio-Rad Laboratories, Hercules, CA, USA). The mycobacterial antigens were prepared as previously described [20]. In short, mycobacteria were grown as a surface pellicle on liquid Reids or Sauton medium. Proteins secreted by the bacteria into the culture medium were precipitated using ammonium sulphate, dissolved in PBS, dialysed and sterile filtrated (0.2 µm). Recombinant MAP antigens and pooled peptides from single MAP antigens used for testing are listed in Table S1. Synthetic peptides were purchased from Genscript, NJ, USA.

### Antibodies

The following antibodies were used for analyzing T cells by flow cytometry: anti-TCR αβ FITC (IgM, clone T10B9.1A-31), anti-CCR6 PE (IgG1κ, clone 11A9), anti-TCRγδ APC (IgG1κ, clone B1), anti- IFNγ FITC (IgG1κ, clone 4S.B3) isotype control FITC (IgM, clone G155-228) isotype control PE (IgG1κ, clone MOPC-21) (all BD Pharmingen); anti-CD4 PE (IgG2a, clone EDU-2), anti-CD8 FITC (IgG2a, clone UCHT-4), Isotype PE (IgG2A, clone BH1), Isotype FITC (IgG2A, clone BH1) (all Diatec) and anti-IL-17a Alexa fluor647 (IgG1κ, clone eBio64DEC17) (ebioscience). Anti-CD56 (IgG2a, clone MEM 188) and goat anti-mouse-IgG2a FITC (Southern Biotechnology).

The HLA restriction of the T cells was determined by testing inhibition of T-cell proliferation in the presence of monoclonal antibodies B8.11 (pan-DR), SPV-L3 (pan-DQ) or B7/21 (pan-DP) at a concentration of 20 µg/ml.

### Establishment of T-cell lines and clones

The protocol for establishment of T-cell lines was adapted from the protocol used for establishing T-cell lines from small intestinal biopsies of celiac disease patients [21], [22] with some modifications. The biopsies were taken from the distal part of the small intestine, or upper part of colon. In patients with active disease, biopsies were taken from inflamed mucosa and from the surrounding non-inflamed areas. Separate, single biopsy specimens from each location were incubated with either complete medium (RPMI 1640 (Gibco) containing 10% human serum, β mercaptoethanol, penicillin, streptomycin and fungizone) or complete medium with MAP (100 µg/ml) overnight. After incubation the biopsies were homogenized for 120 seconds in a BD Medimachine Medicon (BD Medimachine™ Medicon, 35 µm Sterile). The single cells from each biopsy were centrifuged, dissolved in 1 ml complete medium containing 2×10^6^ autologous, irradiated (25 Gy) PBMC, 10 U/mL human IL-2 (R&D Systems, Abingdon, UK), and 1 ng/mL human IL-15 (R&D Systems) and seeded into 8 wells on a U-bottomed 96-well plate. On day 8, cells from duplicate wells were restimulated separately with 1×10^6^ allogenic, irradiated PBMC, 10 U/mL IL-2, 1 ng/mL IL-15, and 1 µg/mL phytohemagglutinin (Remel) in a 48-well plate and propagated as four separate T-cell lines. The four lines established from each biopsy were tested in triplicates on day 15.

T-cell clones were generated from MAP and *E. coli* reactive biopsy-derived T-cell lines. The T cells were diluted in irradiated feeders from three donors with IL-2, IL-15 and PHA as described above and seeded on Terasaki plates (Greiner Bio-One) at a concentration of 0.3–3 cells/well. After 9 days, growing T-cell clones were transferred to 48 well plates and restimulated as before. Established T-cell clones were tested for reactivity to MAP or *E. coli*. T-cell clonality was tested by the IOTest® Beta Mark (Beckman Coulter) TCR Vβ staining kit covering about 70% of the normal human TCR Vβ repertoire of CD3^+^ lymphocytes.

### APC and T cell proliferation assay

Testing of the reactivity of the T-cell lines was done by assessing proliferation in restimulation assays using irradiated adherent cells as APC. The APC were isolated by incubating PBMC (50 000 cells/well, 96 well plate) in medium containing 15% FCS for 1.5 hours. The wells were washed three times in medium with 15% FCS before medium with 10% human serum and antigens were added. The plates were irradiated (25 Gy) the next day before the T cells were added. Autologus adherent cells from frozen PBMC were used for all initial screenings while HLA-II matched donors or autologus APC were used for later testing. The cells were incubated for three days with the addition of ^3^H thymidine for the last 20 hours. Proliferation was assessed by scintillation counting after harvesting of the cultures. Positive T-cell responses were defined as a stimulatory index (SI) above 5 ([T+APC+antigen] divided by [T+APC]).

The following homozygous B-lymphoblastoid cell lines derived from the 10^th^ and 11^th^ International Histocompatibility Workshop (IHWS) were used as APC for identification of HLA restriction of the MAP1508 specific T-cell clone: #9002/MZ070782; (DRB1*0102, DQA1*0101, DQB1*0501, DPB1*0401), #9003/KAS116; (DRB1*0101, DQA1*0101, DQB1*0501, DPB1*1301), #9042/TISI; (DRB1*1103, DQA1*0501, DQB1*0301, DPB1*0402), #9055/H0301; (DRB1*1302, DQA1*0102, DQB1*0605, DPB1*0501), #9063/WT47; (DRB1*1302, DQA1*0102, DQB1*0604, DPB1*1601) and YT (DRB1*0405, DQA1*03, DQB1*0401, DPB1*0501). The B-cell lines were all irradiated with 75 Gy before use.

### Staining of intracellular cytokines

T-cell clones (approximately 500 000 cells) were stimulated with PMA (10 ng/ml) and ionomycin (2 µM) or left unstimulated. Monensin was added and the cells were incubated for 18 hours followed by staining for intracellular IFN-γ and IL-17. Briefly the cells were fixed in 1% PFA for one hour and permabilized in PBS with 2% FCS and 0.2% saponin for 30 minutes. The cells were stained with antibodies against IFN-γ and IL-17a and analyzed on a FACS CALIBUR flow cytometer (Becton Dickinson), equipped with Cell-Quest software.

### Cytokine assays

The amount of cytokines was measured in supernatant from antigen stimulated T-cell clones and unstimulated controls. The stimulation was performed as for the T-cell proliferation assay, and the supernatant was removed after 48 hours and stored at −20C until tested. As a control for T-cell viability ^3^H thymidine was added and the T-cell proliferation measured after incubation for another 24 hours. The amount of cytokines was measured using the Bio-plex™ Cytokine Assay, (Bio-Rad) according to the manufacturer's instructions. Values above the detection limits defined by the standard curve were considered positive.

### Statistics

The Wilcoxon Mann-Whitney non-parametric test was used to compare patient groups and p<0.05 was considered significant.

## Results

### Reactivity of intestinal T cells to various bacterial antigens

The T-cell lines generated without *ex vivo* stimulation with any antigen, were tested for responses in a T-cell proliferation assay against antigen preparations from *B. thetaiotaomicron*, *L. gasseri*, *B. bifidum, E. coli* and MAP. Minimal responses were seen in patients with inactive disease and in non-IBD patients while the results from CD patients with active disease are summarized in Table 2. T-cell lines that reacted to MAP were detected in 5 of 7 (71%) patients in the CD group. Next to MAP, responses to *E. coli* were most frequently detected, and one CD patient (CD-47) had a strong response to *E. coli* with no response to MAP. More T-cell lines reacted to MAP than to the other bacterial antigens in the CD group, while in the UC group there were similar responses to MAP and the commensal bacteria (Table 3). Some of the T-cell lines, especially in the CD group, exhibited extensive multi-reactivity with response to several of the tested antigen preparation. Whether the multi-reactivity was due to cross reactive T cells or the presence of multiple specificities in the T-cell lines was not investigated. There were no systematic differences between T-cell lines obtained from biopsies from inflamed area and biopsies taken from the surrounding non-inflamed area.

**Table 2:** Response to various bacteria in T-cell lines from CD patients with active disease.

	Number of positive^A^ T-cell lines (n = 8)				
	MAP	*B. thetaiotaomicron*	*B. bifidum*	*L. gasseri*	*E. coli*
CD-9	5 (2)^B^	1 (1)	2 (2)	2 (2)	1 (1)
CD-18	5 (2)	1 (1)	0	0	2 (2)
CD-15	7 (0)	0	0	0	0
CD-48	2 (0)	0	1 (1)	1 (1)	0
CD-46	2 (2)	1 (1)	1 (1)	ND^C^	3 (2)
CD-47	0	1 (1)	0	0	3 (1)
CD-10	0	0	0	0	0

A Positive lines are defined as SI>5

B Some lines reacted to several bacterial antigens. The total number of positive lines to each bacterium is shown. The number in brackets shows how many of these lines that were multi-reactive.

C ND = not done

**Table 3:** Reactivity of T-cell lines to MAP and commensal bacteria in CD and UC patients with active disease

	Number of positive^A^ T-cell lines (n = 8)		
	MAP^B^	Multi-reactive C	Commensal^D^
**CD patients**			
CD-9	**3**	**2**	0
CD-18	**3**	**2**	0
CD-15	**7**	0	0
CD-48	**2**	0	**1**
CD-46	0	**2**	**1**
CD-47	0	0	**3**
CD-10	0	0	0
**UC patients**			
UC-27	**4**	0	0
UC-40	**1**	0	0
UC-14	0	0	0
UC-31	0	**1**	**3**
UC-32	0	0	**1**
UC-45	0	0	**1**
UC-44	0	0	0
UC-16	0	0	0

A Positive lines are defined as SI>5

B Lines reacted only with MAP.

C Lines reactive with MAP and one or more commensal bacteria

D Lines reactive to one or more of the following commensal bacteria: *B. thetaiotaomicron*, *L. gasseri*, *B. bifidum*, *E. coli*

**Figure 1:**
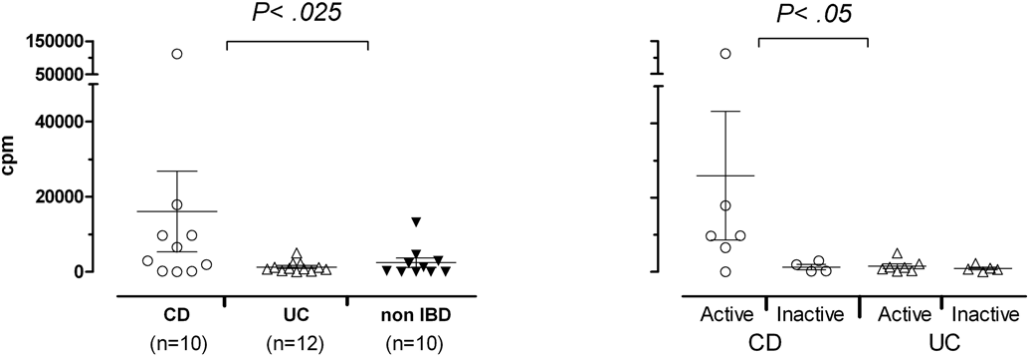
T-cell responses to MAP. Autologous adherent cells were incubated with antigen overnight and T cells were added followed by incubation for three days. ^3^H thymidine was added for the last 20 hours, and proliferation was assessed by scintillation counting after harvesting of the cultures. A) Depiction of results from all patients. B) Depiction of the same results, but where the material is divided into subgroups with active and inactive disease; active CD; n = 6, inactive CD; n = 4, active UC; n = 7, inactive UC; n = 5. Each symbol represents the mean response in four T-cell lines made from one patient. The lines were screened in triplicates and the results are given as CPM [ (T+ APC + MAP)−(T+APC)]. There was a significant difference between CD and UC patients (p<0.025) and between patients with active and inactive CD (p<0.05) using the non-parametric Wilcoxon Mann-Whitney test. Error bars indicate the mean response in the group±SEM.

Next we wanted to enrich for mycobacteria reactive T cells, and lines were therefore also generated from intestinal biopsies stimulated with MAP *ex vivo*. These lines were tested for response to MAP. The MAP stimulated biopsies from non-inflamed mucosa of one CD patient and one UC patient with active disease were contaminated and disregarded. The mean response in T-cell lines generated from non-inflamed mucosa of CD patients was significantly (p<0.025) higher than the responses in UC patients (Figure 1A). A similar tendency was seen in T cells from inflamed mucosa (not statistically significant, data not shown). The responses were strongest in CD patients with active disease compared to CD patients with inactive disease (p<0.05) (Figure 1B). Proliferation was however also detected in T-cell lines from some of the patients with inactive CD and in some control patients. These patients usually had responses in one single line T-cell line while patients with active CD had responses in several T-cell lines suggesting a higher frequency of MAP reactive T cells in the latter group. The three CD patients (CD-10, CD-33 and CD-36) with no detectable response to MAP in any of the tested T-cell lines had colon involvement only (Table 1).

### Cytokine response in MAP and E. coli responsive T-cell clones

In addition to MAP, *E. coli* was the bacterial antigen eliciting the strongest responses in CD patients.To further characterize the T cells in CD patients, we attempted to isolated single T-cell clones from three patients with strong MAP responses, one patient with a strong *E. coli* response and one patient with a mixed MAP and *E. coli* response. Altogether we obtained 28 T-cell clones (CD-46:17 clones, CD-11: 3 clones, CD-6: 2 clones and CD-9: 6 clones) that reacted to MAP antigens. From patient CD-46, who had a mixed response we also obtained eight *E. coli* reactive T-cell clones. From patient CD-47 with a strong *E. coli* response, a loss of reactivity was seen after expansion of the T-cell line and cloning was thus abandoned.

**Figure 2:**
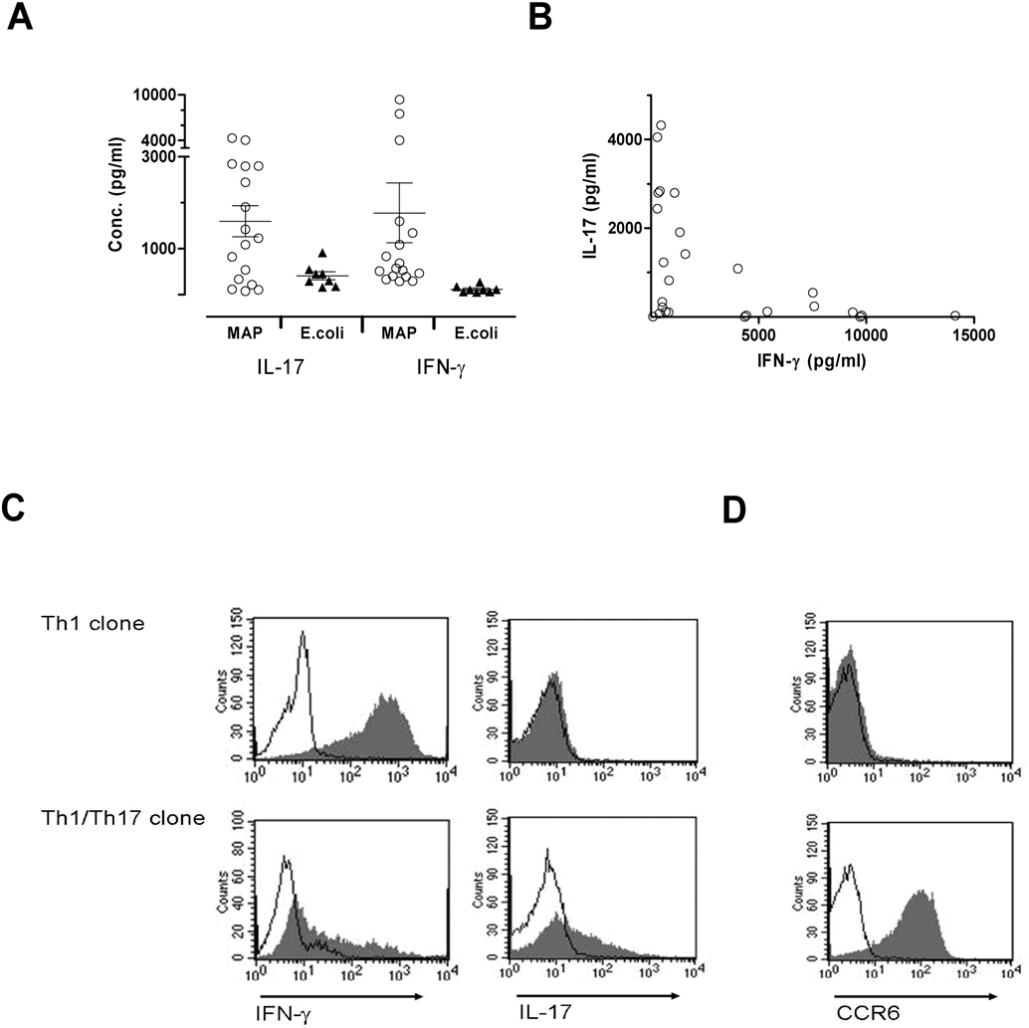
Cytokine responses in T-cell clones. A) Comparison of cytokine response in MAP reactive (n = 17) and *E. coli* reactive (n = 8) T-cell clones from the same CD patient (CD-46). T-cell clones were stimulated with MAP or *E. coli* antigens (10 µg/ml) for 48 hours using HLA class II matched irradiated adherent cells as APC. Supernatants from duplicate wells were sampled and tested for cytokine production. Cytokine production in control wells was subtracted. One symbol represents one clone. Error bars indicate mean±SEM. B) IL-17 and IFN-γ production in MAP reactive T-cell clones (n = 26) from four different CD patients in response to MAP antigen. C) Intracellular staining of IFN-γ and IL-17 after stimulation with PMA/ionomycin in a Th1 (TCC906.A.8.4.15) clone and a Th1/Th17 clone (TCC946.A.8.2b.17). Filled histogram represent PMA/ionomycin samples and open histograms represent unstimulated samples. D) CCR6 expression in a Th1 clone and (top) and a Th1/Th17 clone (bottom). Filled histograms represent CCR6 expression and open histograms represent isotype control.

CD patients have a granulomatous inflammation with excessive production of IL-17 and IFN-γ in the intestine [16]. We thus wanted to see whether the MAP reactive and the *E. coli* reactive T-cell clones secreted any of these inflammatory cytokines. Two MAP reactive T-cell clones could not be expanded and thus excluded from further studies. The T-cell clones were incubated with MAP (n = 26) or *E. coli* (n = 8) antigens using HLA-II matched adherent cells as APCs and the supernatant was assayed for IFN-γ, IL-17 and IL-4. All the MAP reactive clones produced IFN-γ (ranging from 88 to 9786 pg/ml) and 23 of 26 clones produced IL-17 (ranging from 25 to 4320 pg/ml), while low levels of IL-4 (<17 pg/ml) was detected in three clones. The *E. coli* reactive clones produced some IL-17 (411 pg/ml±81) and lower, but detectable amounts of IFN-γ (117 pg/ml±25). In comparison the MAP reactive clones (n = 17) from the same patient produced a mean of 1593 pg/ml±328 of IL-17 and 1770 pg/ml±631 of IFN-γ (Figure 2A). Although most of the MAP reactive clones produced both IFN-γ and IL-17 they appeared to have either a dominant IL-17 secretion or a dominant IFN-γ production (Figure 2B). To see if polyclonal activation would give a different cytokine pattern, three clones producing only IFN-γ and two clones producing predominantly IL-17 but with detectable IFN-γ in response to MAP, were stimulated with PMA/ionomycin. The results were comparable to what was seen after antigen stimulation of the same clones. No IL-17 was detected in the IFN-γ secreting clones while the IL-17 producing clones made both cytokines (Figure 2C). This suggested that MAP reactive clones with a Th1 and a Th1/Th17 mixed phenotype were present in CD patients. A typical marker of IL-17 producing cells is the chemokine receptor CCR6, and all but one T-cell clone expressed CCR6 (Figure 2D). The CCR6 negative clone, TCC906A.8.4.15, had a Th1 phenotype with no detectable IL-17 in response to MAP or PMA/ionomycin.

### MAP reactive T-cell clones from CD patients showed a dominant response to the M. avium-intracellulare complex

**Figure 3:**
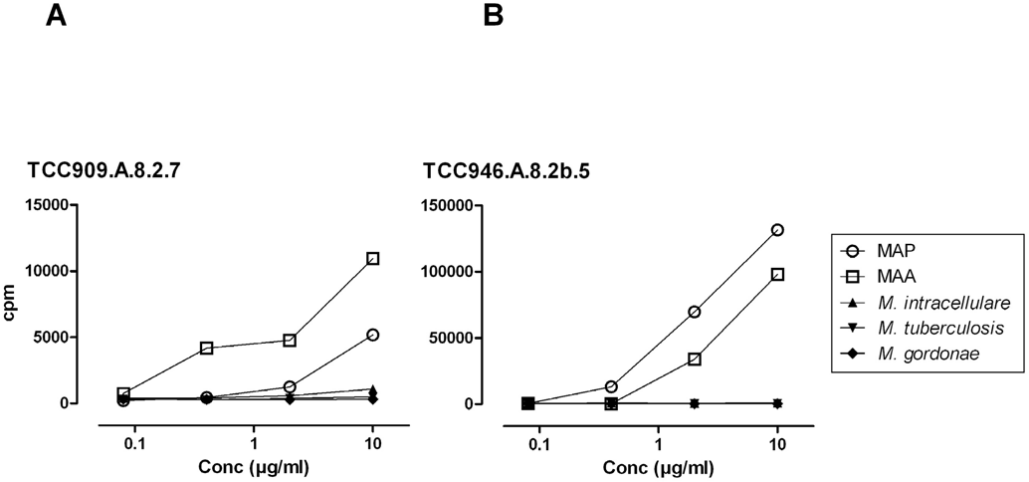
Responses of T-cell clones to various mycobacteria. Proliferation of T-cell clones from two different CD patients (CD-9 and CD-46) after stimulation with crude antigen preparations from various mycobacteria. HLA-II matched adherent cells were used as APC and were incubated with antigen overnight. The T cells were added followed by incubation for three days. ^3^H thymidine was added for the last 20 hours. Each concentration of antigen was tested in duplicates. Most T-cell clones showed some degree of cross-reactivity to mycobacterial antigens. A) Depiction of results of one clone (TCC909.A.8.2.7) which responded to MAP, MAA and *M. intracellulare* B) Depiction of results of one clone (TCC946.A8.2b.5) which responded only to MAP and MAA. The data are representative for three independent experiments.

MAP share several highly cross-reactive antigens with other mycobacteria, and exposure to environmental mycobacteria could lead to detectable T-cell responses. The T-cell clones were subsequently screened for reactivity to various crude antigen preparations from different mycobacterial species. Most of the T-cell clones showed some degree of cross-reactivity, however one clone (TCC946.A.8.2b.5 from CD-46) responded only to MAP and the very closely related *Mycobacterium avium* subspecies *avium* MAA (Figure 3). Another clone (TCC909A.8.2.7) from a different patient (CD-9) responded to MAP, MAA and had a low, but detectable response to *Mycobacterium intracellulare*. The percentages of MAP reactive T-cell clones responding to the other mycobacteria were, MAA 100%, *M. intracellulare* 92%, *M. gordonae* 65% and *M. tuberculosis* 31%.

### HLA restriction

**Figure 4:**
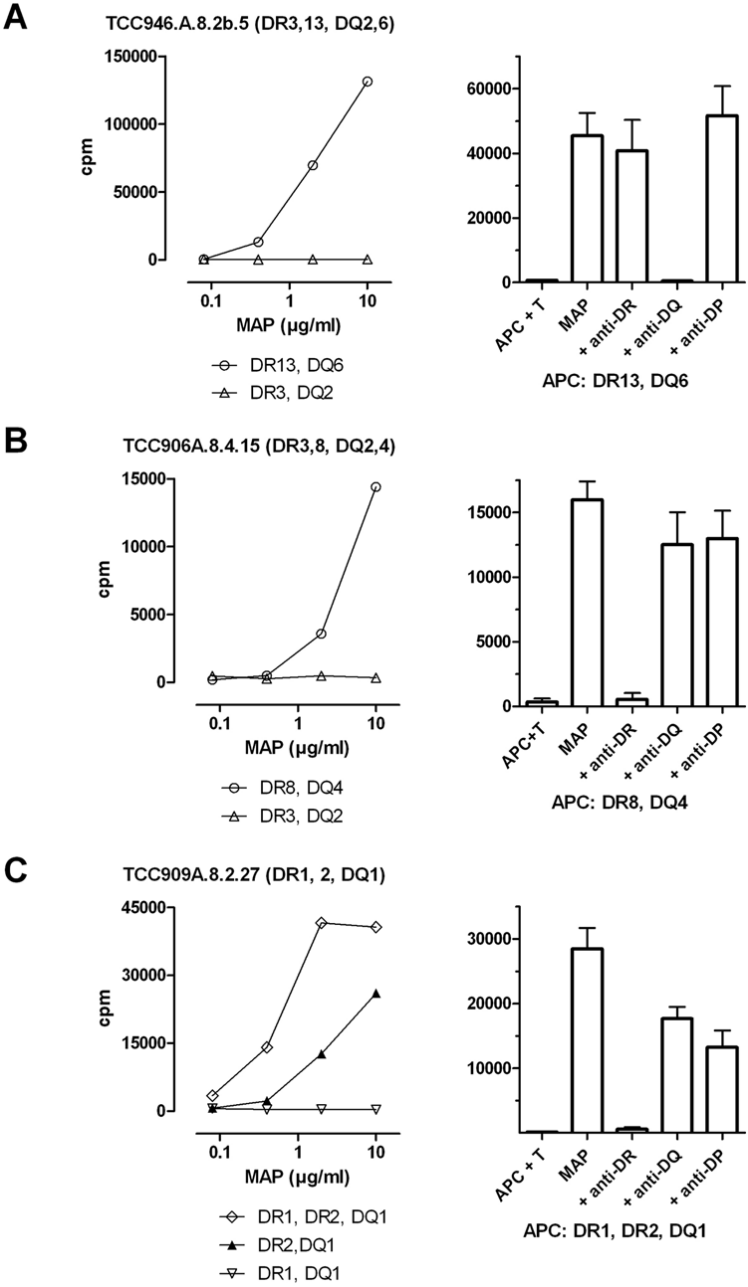
HLA restriction of T-cell clones. A) Patient CD-46, B) Patient CD-6 and C) Patient CD-9. The individual patients HLA DR/DQ serotype of the clone donor is given in brackets after the clone identification tag. Adherent cells from HLAII DR/DQ haplotype matched donors were used as APC and incubated with antigen overnight. T cells were added followed by further incubation for three days with addition of ^3^H thymidine for the last 20 hours. Left panel depicts responses using different APC. Right panel depicts blocking of the responses by addition of monoclonal antibodies specific for either HLA-DR, HLA-DQ or HLA-DP two hours prior to addition of the T cells. The blocking assay was done in triplicates and repeated three times. Error bars indicate mean±SD.

The T-cell restriction was determined using APCs from DR/DQ haplotype matched donors together with blocking of the response in a T-cell assay by adding specific anti-HLA-DP, anti-HLA-DQ and anti-HLA-DR antibodies (Figure 4). The MAP reactive clones from two of the patients (CD-6 and CD-9) were DR restricted (n = 7) while DQ restricted clones (n = 18) were obtained from the two others (CD-11 and CD-46). The response could be blocked in all clones except three. These clones were CD4+, TCRαβ+, CD56- and appeared to be conventional T cells. One could speculate that they might recognize antigen in the context of CD1 which is seen in other mycobacterial infections. However, this was not pursued in the present study.

### Characterization of a T-cell clone responding to MAP1508

**Figure 5:**
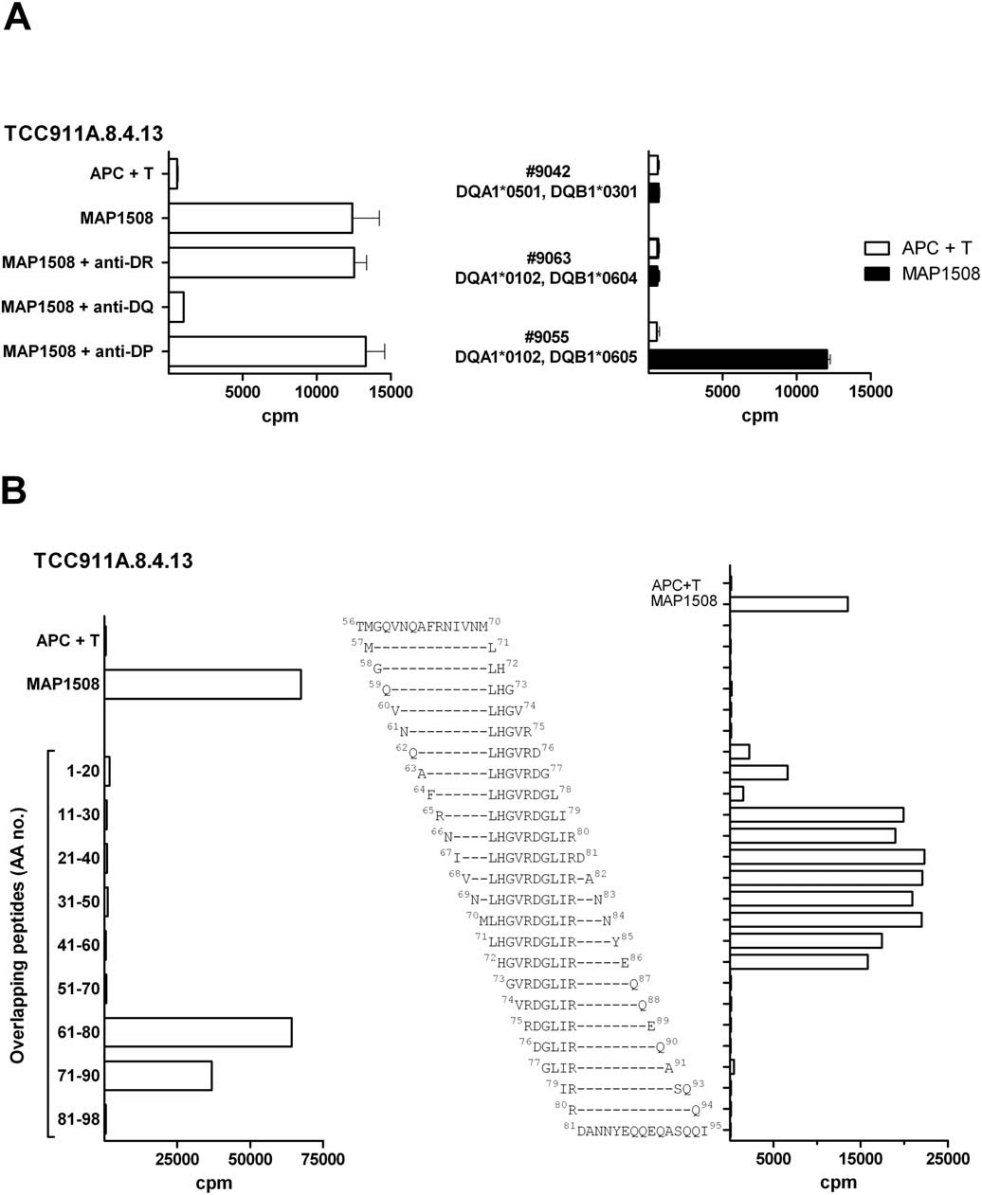
Epitope mapping of a MAP1508 specific T-cell clone. A) HLA-II restriction of TCC911.A.8.4.13. Left; blocking of responses by HLA class II specific monoclonal antibodies. Right; HLA-DQ matched EBV cells were used as APC to identify HLA restriction. The patient was DQB1*0609 and DQB1*0301. Error bars indicate mean±SD. B) Proliferation of the T-cell clone in response to overlapping peptides (10 µM) of the MAP1508 protein. Left; peptides of 20 aa with10 aa overlap. Right; peptides of 15 aa overlapping with one aa ranging from position 56 to 95. The sequences are shown.

Finally we aimed to identify which antigen in the crude MAP preparation the T cells responded to. T-cell lines and a selection of T-cell clones were thus tested for responses against a range of available recombinant purified MAP antigens or pools of overlapping peptides (Table S1). One T-cell line had a strong response to pooled peptides from MAP1508 [23] which is 87% identical to esxP from *M. tuberculosis*. By cloning of this line we isolated a T-cell clone (TCC911.A.8.4.13) responding to this antigen. The T-cell clone recognized the peptides in the context of HLA-DQ as showed by adding anti-HLA-DP, anti-HLA-DQ or anti-HLA-DR antibodies (Figure 5A). The patient was DQB1*0609 and DQB1*0301. HLA-DQ matched EBV cells were used as APC, and the results indicated that this clone recognized the peptide in the context of DQ6 (i.e. DQA1*0102/DQB1*0605). The clone did not recognize the peptide in the context of DQA1*0102/DQB1*0604, which might be due to a histidine at position 30 in DQB1*0604 compared to a tyrosine in DQB1*0605 and DQB1*0609. Epitope mapping demonstrated that the epitope was located at aa position 71-80 of MAP1508 (Figure 5B). Protein Blast using these 10 aa confirmed that the epitope is conserved in several of the pathogenic mycobacteria including the *M. avium* complex and the *M. tuberculosis* complex, while it was not found in the non-pathogenic *Mycobacterium smegmatis* mc^2^ 155. Staining for TCR Vβ showed that the clone expressed the TCR Vβ8 chain. Furthermore, the clone was CD4+, Tαβ+ CCR6+, and it produced IFN-γ and not IL-17 in response to PMA/ionomycin (data not shown). The T cells from the other patients did not recognize any of the available purified antigens.

## Discussion

This study demonstrated that T cells reacting to various bacteria were present in the intestine of patients with CD, however a majority of the patients had a dominant response to MAP. The T cells secreted IFN-γ and IL-17, and a role for mycobacteria in the excessive inflammation seen in CD cannot be excluded. The isolation of T cells together with identification of their specificity is a useful approach to get answers about the relative importance of various bacteria in CD.

Although the CD lesions have increased number of CD4 T cells producing inflammatory cytokines, very few studies have focused on the specificity of these intestinal T cells. Duchmann et al published two studies that showed the presence of T cells responding to commensal bacteria in equal frequencies in UC and CD patients with active disease [17], [18], which is in agreement with our findings. Interestingly, we found that intestinal T cells from CD patients responded more vigorously to MAP antigens compared with T cells from UC patients. There was also an apparently higher frequency of MAP reactive T cells compared with T cells responding to commensal bacteria in the CD patients while no such difference was found in UC patients. MAP causes a disease in ruminants with similarities to CD and the bacterium has been found in higher frequencies in CD patients compared to controls [14], [15]. However, convincing data showing a cellular immune response to MAP are lacking. Some studies have looked into T cell responses against mycobacteria in CD patients, however the methodology in the current work is vastly different. Previous studies have largely used PBMC and/or looked at antigen induced suppression [24], [25]. To the best of our knowledge this is the first study where isolated intestinal T cells were used to investigate cellular immune responses to MAP in CD patients.

All the T-cell clones in the present study produced IL-17 and/or IFN-γ. T cells that produced the inflammatory cytokines IFN-γ and IL-17 have been shown to be increased in the intestine of CD patients [16], but the specificity of any of these Th1/Th17 clones has not previously been identified. The genetic associations of *NOD2* and *IL23R* with CD suggest that IL-17 is relevant for disease development. It has been shown that IL-23 induced IL-17 production from memory T cells [26] and that stimulation of NOD2 promoted IL-17 production through a synergistic effect of IL-23 and IL-1 [27] . The ligand for NOD2 is known to be muramyl dipeptide (MDP) which is present in the cell wall of bacteria. However, it is recognized that most bacterial species produce only N-acetyl-MDP, in contrast to mycobacteria which also produce N-glycolyl MDP [28], [29]. Studies comparing these forms of MDP have shown that N-glycolyl MDP is more potent than N-acetyl MDP at inducing NOD2-dependent pro-inflammatory responses (Behr, personal communication). Furthermore an association between NOD2 polymorphism and resistance to MAP in cattle has been described [30]. Altogether these recent studies suggest a link between mycobacteria, NOD2 and the IL-23/IL-17 pathway and are in agreement with our findings. It cannot be excluded that the method used to isolate the T-cell clones influenced their cytokine profile, and a future challenge is to confirm that the MAP reactive T cells also produce IL-17 *in vivo*.

In the present study there was a strong T-cell response to MAP in the CD group, but some of the control patients also showed some reactivity. This was not surprising considering that a crude mycobacterial antigen preparation was used. Mycobacteria contain several antigens with high degree of homology, and humans can be exposed to a range of environmental mycobacteria that might trigger an immune response. In addition, the Norwegian population is vaccinated with Bacille Calmette Guerin (BCG) and one could speculate that BCG reactive T cells can be found at the site of inflammation in IBD patients. Although this cannot be totally excluded, it is not likely to be a major confounding factor. The majority (69%) of the T-cell clones did not respond to *M. tuberculosis.* BCG is attenuated from *Mycobacterium bovis* by deletion of several genetic regions, and all of the genes in this vaccine strain are also present in *M. tuberculosis* [31], [32]. Consequently, BCG reactive T cells are likely to cross-react with antigens from *M. tuberculosis*. To find conclusive evidence that the responses were caused by MAP is difficult since proteins from MAP and MAA have a extremely high degree of identity [23]. However, we isolated one T-cell clone that responded only to MAP and MAA, while another clone from another patient responded to MAA, MAP and *M. intracellulare.* MAP and MAA are both subspecies of *M. avium* while *M. intracellulare* is the mycobacterial species that is phylogenetically closest to *M. avium*. Of these bacteria, MAP is the only organism that has a predilection for the intestinal mucosa while MAA and *M. intracellulare* usually causes cervical lymphadenitis in children or also disseminated or pulmonary disease particularly in immunocompromised individuals. These findings suggest that at least in two of the patients the responses were triggered by MAP or a closely related bacterium belonging to the *M. avium* complex.

A future challenge is to identify how many, and which patients have a MAP or an *M. avium* complex specific T-cell response. T-cell cloning is tedious and not an option for screening of large number of patients. An alternative is to use MAP specific epitopes and test for recognition of these in polyclonal T cell lines derived from intestinal biopsies of patients carrying the relevant HLA class II restriction element. Identification of such epitopes is challenging, but may be achieved using a panel of MAP specific T-cell clones to screen peptide libraries or MAP expression libraries. These methods have previously been used successfully to identify the epitopes of T cells of unknown specificity [33]–[35]. To date we have identified the specificity of one T-cell clone. The epitope was located on an esx protein which belongs to the highly immunogenic ESAT family [36]. The epitope was conserved in several pathogenic mycobacterial species, but not found in the genome of the saprophytic *M. smegmatis*. Further studies will focus on identification of MAP specific epitopes that can be used to screen T-cell lines from a larger number of CD patients and controls

CD presents with a variety of clinical manifestation and genes associated with CD differs between populations. It is thus possible that certain bacteria can be of importance in a subgroup of patients.We believe that the isolation of tissue derived T cell clones followed by characterization of their specificity can give novel answers about the bacteria involved in the inappropriate inflammatory response seen in CD. This study demonstrated the presence of MAP reactive intestinal T-cell clones producing IFN-γ and IL-17 suggesting that they may contribute to the intestinal inflammation.

## Supporting Information

Table S1 Recombinant antigens and pooled peptides used in the present study
(0.06 MB DOC)

## References

[1] Baumgart DC, Carding SR (2007). Inflammatory bowel disease: cause and immunobiology.. Lancet.

[2] Ogura Y, Bonen DK, Inohara N, Nicolae DL, Chen FF (2001). A frameshift mutation in NOD2 associated with susceptibility to Crohn's disease.. Nature.

[3] Hugot JP, Chamaillard M, Zouali H, Lesage S, Cezard JP (2001). Association of NOD2 leucine-rich repeat variants with susceptibility to Crohn's disease.. Nature.

[4] Hampe J, Franke A, Rosenstiel P, Till A, Teuber M (2007). A genome-wide association scan of nonsynonymous SNPs identifies a susceptibility variant for Crohn disease in ATG16L1.. Nat Genet.

[5] Parkes M, Barrett JC, Prescott NJ, Tremelling M, Anderson CA (2007). Sequence variants in the autophagy gene IRGM and multiple other replicating loci contribute to Crohn's disease susceptibility.. Nat Genet.

[6] Eckmann L, Karin M (2005). NOD2 and Crohn's disease: loss or gain of function?. Immunity.

[7] Strober W, Fuss I, Mannon P (2007). The fundamental basis of inflammatory bowel disease.. J Clin Invest.

[8] Cadwell K, Liu JY, Brown SL, Miyoshi H, Loh J (2008). A key role for autophagy and the autophagy gene Atg16l1 in mouse and human intestinal Paneth cells.. Nature.

[9] Rich KA, Burkett C, Webster P (2003). Cytoplasmic bacteria can be targets for autophagy.. Cell Microbiol.

[10] Rioux JD, Xavier RJ, Taylor KD, Silverberg MS, Goyette P (2007). Genome-wide association study identifies new susceptibility loci for Crohn disease and implicates autophagy in disease pathogenesis.. Nat Genet.

[11] Singh SB, Davis AS, Taylor GA, Deretic V (2006). Human IRGM induces autophagy to eliminate intracellular mycobacteria.. Science.

[12] Barrett JC, Hansoul S, Nicolae DL, Cho JH, Duerr RH (2008). Genome-wide association defines more than 30 distinct susceptibility loci for Crohn's disease.. Nat Genet.

[13] Boudeau J, Glasser AL, Masseret E, Joly B, Darfeuille-Michaud A (1999). Invasive ability of an *Escherichia coli* strain isolated from the ileal mucosa of a patient with Crohn's disease.. Infect Immun.

[14] Feller M, Huwiler K, Stephan R, Altpeter E, Shang A (2007). *Mycobacterium avium* subspecies *paratuberculosis* and Crohn's disease: a systematic review and meta-analysis.. Lancet Infect Dis.

[15] Abubakar I, Myhill D, Aliyu SH, Hunter PR (2008). Detection of *Mycobacterium avium* subspecies *paratuberculosis* from patients with Crohn's disease using nucleic acid-based techniques: a systematic review and meta-analysis.. Inflamm Bowel Dis.

[16] Annunziato F, Cosmi L, Santarlasci V, Maggi L, Liotta F (2007). Phenotypic and functional features of human Th17 cells.. J Exp Med.

[17] Duchmann R, Marker-Hermann E, Meyer zum Buschenfelde KH (1996). Bacteria-specific T-cell clones are selective in their reactivity towards different enterobacteria or *H. pylori* and increased in inflammatory bowel disease.. Scand J Immunol.

[18] Duchmann R, May E, Heike M, Knolle P, Neurath M (1999). T cell specificity and cross reactivity towards enterobacteria, *Bacteroides*, *Bifidobacterium*, and antigens from resident intestinal flora in humans.. Gut.

[19] Lowry OH, Rosebrough NJ, Farr AL, Randall RJ (1951). Protein measurement with the Folin phenol reagent.. J Biol Chem.

[20] Olsen I, Reitan LJ, Holstad G, Wiker HG (2000). Alkyl hydroperoxide reductases C and D are major antigens constitutively expressed by *Mycobacterium avium* subsp. *paratuberculosis*.. Infect Immun.

[21] Molberg O, Solheim FN, Jensen T, Lundin KE, rentz-Hansen H (2003). Intestinal T-cell responses to high-molecular-weight glutenins in celiac disease.. Gastroenterology.

[22] Molberg O, McAdam S, Lundin KE, Sollid LM, Marsh MN (2000). Studies of Gliadin-Specific T-cells in Celiac Disease.. Celiac Disease.

[23] Li L, Bannantine JP, Zhang Q, Amonsin A, May BJ (2005). The complete genome sequence of *Mycobacterium avium* subspecies *paratuberculosis*.. Proc Natl Acad Sci U S A.

[24] Seldenrijk CA, Drexhage HA, Meuwissen SG, Meijer CJ (1990). T-cellular immune reactions (in macrophage inhibition factor assay) against *Mycobacterium paratuberculosis, Mycobacterium kansasii, Mycobacterium tuberculosis, Mycobacterium avium* in patients with chronic inflammatory bowel disease.. Gut.

[25] Dalton HR, Hoang P, Jewell DP (1992). Antigen induced suppression in peripheral blood and lamina propria mononuclear cells in inflammatory bowel disease.. Gut.

[26] Aggarwal S, Ghilardi N, Xie MH, de Sauvage FJ, Gurney AL (2003). Interleukin-23 promotes a distinct CD4 T cell activation state characterized by the production of interleukin-17.. J Biol Chem.

[27] van Beelen AJ, Zelinkova Z, Taanman-Kueter EW, Muller FJ, Hommes DW (2007). Stimulation of the intracellular bacterial sensor NOD2 programs dendritic cells to promote interleukin-17 production in human memory T cells.. Immunity.

[28] Azuma I, Thomas DW, Adam A, Ghuysen JM, Bonaly R (1970). Occurrence of N-glycolylmuramic acid in bacterial cell walls. A preliminary survey.. Biochim Biophys Acta.

[29] Raymond JB, Mahapatra S, Crick DC, Pavelka MS (2005). Identification of the namH gene, encoding the hydroxylase responsible for the N-glycolylation of the mycobacterial peptidoglycan.. J Biol Chem.

[30] Pinedo PJ, Buergelt CD, Donovan GA, Melendez P, Morel L (2009). Association between CARD15/NOD2 gene polymorphisms and paratuberculosis infection in cattle.. Vet Microbiol.

[31] Behr MA, Wilson MA, Gill WP, Salamon H, Schoolnik GK (1999). Comparative genomics of BCG vaccines by whole-genome DNA microarray.. Science.

[32] Garnier T, Eiglmeier K, Camus JC, Medina N, Mansoor H (2003). The complete genome sequence of *Mycobacterium bovis*.. Proc Natl Acad Sci U S A.

[33] Hemmer B, Gran B, Zhao Y, Marques A, Pascal J (1999). Identification of candidate T-cell epitopes and molecular mimics in chronic Lyme disease.. Nat Med.

[34] Hiemstra HS, Duinkerken G, Benckhuijsen WE, Amons R, De Vries RR (1997). The identification of CD4+ T cell epitopes with dedicated synthetic peptide libraries.. Proc Natl Acad Sci U S A.

[35] Coler RN, Dillon DC, Skeiky YA, Kahn M, Orme IM (2009). Identification of *Mycobacterium tuberculosis* vaccine candidates using human CD4(+) T-cells expression cloning.. Vaccine.

[36] van Pinxteren LA, Ravn P, Agger EM, Pollock J, Andersen P (2000). Diagnosis of tuberculosis based on the two specific antigens ESAT-6 and CFP10.. Clin Diagn Lab Immunol.

