# Supplementary material for: Isolation of Mycobacterium avium Subspecies paratuberculosis Reactive CD4 T Cells from Intestinal Biopsies of Crohn's Disease Patients
Source: PLoS One. 2009 May 22;4(5):e5641. doi: 10.1371/journal.pone.0005641 (PMC2682569; doi:10.1371/journal.pone.0005641)
Supplement: Table S1 — Recombinant antigens and pooled peptides used in the present study (0.06 MB DOC) [file pone.0005641.s001.doc]

Table S1.Recombinant proteins and synthetic peptides.

| Recombinant proteins |  |
| --- | --- |
| MBP-LacZ | Control protein |
| MAP0016c pknB | serine-threonine protein kinase |
| MAP0094 | hypothetical protein |
| MAP0284c | hypothetical protein |
| MAP0326 | 19-kDa protein |
| MAP0573c otsA | probable [alpha],-trehalose-phosphate synthase |
| MAP0790 | Enoyl-CoA hydratase |
| MAP1272c | hypothetical protein |
| MAP1693c | Peptidyl-prolyl cis-trans isomerase |
| MAP1696c hsp 18_1 | 18 kDa antigen |
| MAP1737 mmpS5 | small membrane protein |
| MAP1939c | possible membrane protein |
| MAP2121c | hypothetical protein |
| MAP2239 mmpL4 | large membrane protein |
| MAP2417c lppJ | lipoprotein |
| MAP2663c | integral membrane protein |
| MAP2754 | hypothetical protein |
| MAP2781 | probable muconolactone decarboxylase |
| MAP2973 lppW | Lipoprotein |
| MAP3268 hsp 18_3 | 18 kDa antigen |
| MAP3685c | integral membrane protein |
| MAP3812c | possible membrane protein |
| MAP3890 mmpL4 | large membrane protein |
| MAP3898 | conserved hypothetical protein |
| MAP3906 | probable aminopeptidase Y |
| MAP3920 | tuberculin related peptide (AT103) |
| MAP3938c | 65 kDa antigen |
| MAP3970 | possible exported protein |
| MAP3976 | possible lipoprotein |
| MAP4075c lpqN | lipoprotein |
| MAP4244 | hypothetical protein. |
| MAP4259 | possible membrane protein |
| MAP4288 lpqP | probable esterase |
| MAP4308c | Fructose-bisphosphate aldolase |
|  |  |
| Syntethic peptides |  |
| MAP3784 | esxH (ESAT6 fam) |
| MAP1508 | esxK (ESAT6 fam) |
| MAP4243 | esxU (ESAT6 fam) |
